# Supplementary material for: The Clinical, Microbiological, and Immunological Effects of Probiotic Supplementation on Prevention and Treatment of Periodontal Diseases: A Systematic Review and Meta-Analysis
Source: Nutrients. 2022 Feb 28;14(5):1036. doi: 10.3390/nu14051036 (PMC8912513; doi:10.3390/nu14051036)
Supplement: Supplementary file 1 [file nutrients-14-01036-s001.zip › nutrients-1573684-supplementary.pdf]

The clinical, microbiological and immunological effects of probiotic supplementation on prevention and treatment of periodontal diseases: A systematic review and meta-analysis

Zohre Gheisary, Razi Mahmood, Aparna Harri shivanantham, Juxin Liu, Jessica R.L. Lieffers, Petros Papagerakis and Silvana Papagerakis*

Online Supplementary Material

Contents

Supplemental Table S1. Inclusion & Exclusion Criteria 3

Supplemental Table S2. Search Terms and Strategy 4

Supplemental Table S3: Risk of Bias within Studies 7

Supplemental Table S4. Overall Measure of Effects of Probiotic Supplementation on Clinical, Immunological and Microbiological Outcomes. 8

Subgroup Analysis of the Associations between Probiotic Supplementation and Clinical Outcomes in Periodontal Disease Patients. 10

1. Subgroup Analysis Examining the Effects of Probiotic Supplementation on Plaque Index (P1I) 10

1.A. Type of periodontal disease 10

1.B. Type of probiotic strain 10

1. C. Type of *lactobacillus* species 10

1. D. Treatment duration 10

1. E. Mode of probiotic delivery 10

1. F. Oral hygiene instructions 10

2. Subgroup Analysis Examining the Effects of Probiotic Supplementation on Mean Plaque Percentage (MPP) 10

2. A. Type of periodontal disease 10

2. B. Type of probiotic strain 10

2. C. Type of *lactobacillus* species 11

2. D. Treatment duration 11

2. E. Mode of probiotic delivery 11

2. F. Oral hygiene instructions 11

3. Subgroup Analysis Examining the Effects of Probiotic Supplementation on Gingival Index (GI) 11

3. A. Type of periodontal disease 11

3. B. Type of probiotic strain 11

3. C. Type of *lactobacillus* species 11

3. D. Treatment duration 11

3. E. Mode of probiotic delivery 11

3. F. Oral hygiene instructions 11

4. Subgroup Analysis Examining the Effects of Probiotic Supplementation on Probing Pocket Depth (PPD) 12
4. A. Type of periodontal disease 12
4. B. Type of probiotic strain 12
4. C. Type of *lactobacillus* species 12
4. D. Treatment duration 12
4. E. Mode of probiotic delivery 12
4. F. Oral hygiene instructions 12
4. G. Disease severity 12
4. *H.* Comparison of probiotic and antibiotic use on probing pocket depth 13
5. Subgroup Analysis Examining the Effects of Probiotic Supplementation on Clinical Attachment Level (CAL) 13
5. A. Type of probiotic strain 13
5. B. Type of *lactobacillus* species 13
5. C. Treatment duration 13
5. D. Mode of probiotic delivery 13
5. E. Oral hygiene instructions 13
5. F. Disease severity 13
5. G. Comparison of probiotic and antibiotic use on clinical attachment level 14
6. Subgroup Analysis Examining the Effects of Probiotic Supplementation on Bleeding on Probing (BOP) 14
6. A. Type of periodontal disease 14
6. B. Type of probiotic strain 14
6. C. Type of *lactobacillus* species 14
6. D. Treatment duration 14
6. E. Mode of probiotic delivery 14
6. F. Oral hygiene instructions 14
7. Subgroup Analysis Examining the Effects of Probiotic Supplementation on Gingival Crevicular Fluid (GCF) 15
8. Subgroup Analysis Examining the Effects of Probiotic Supplementation on Gingival Recession (REC) 15
Supplemental Figure S1: Subgroup Analysis Forest Plots Examining the Effects of Probiotic Supplementation on Clinical Outcomes 16

Supplemental Table S1. Inclusion & Exclusion Criteria

	Inclusion	Exclusion
Population	Adults 18 years of age or older, clinically diagnosed with periodontal disease or healthy adults (without periodontal disease).	Studies with children (less than 18 years of age).
Intervention	Probiotic supplementation is the intervention group of the study.	Studies that do not involve probiotic supplementation in the intervention group of the study.
Comparator	Individuals not receiving probiotic supplementation<br>OR<br>Individuals receiving antibiotics	Studies without control groups.
Outcomes	**Clinical outcomes**: Plaque index, gingival index, probing pocket depth, clinical attachment level, bleeding on probing, gingival recession, and gingival crevicular fluid volume.<br><br>**Subgingival Microbiological Count**: *Porphyromonas gingivalis*, *Fusobacterium nucleatum*, *Tannerella forsythia*, *Prevotella intermedia*, *Aggregatibacter actinomycetemcomitans*, *Streptococcus mutans* and *Lactobacillus species*.<br><br>**GCF levels of immunological outcomes**: matrix metalloproteinase-8, interleukin-6, interleukin-1β, interleukin-8, interleukin-10, and tumor necrosis factor-α	Studies that do not assess clinical, microbiological or immunological outcomes.
Study Design	Randomized controlled trials	Any study that is not a randomized controlled trial.
Language	Articles in English	Articles not available in English or with no translation.

Supplemental Table S2. Search Terms and Strategy

Step 1: search for terms relating to periodontal disease	((((periodontal diseases [MeSH Terms]) OR (periodontal disease [MeSH Terms])) OR (“periodontal disease*”[Title/Abstract])) OR (“gingival disease*”[Title/Abstract])
Step 2: search for terms relating to periodontitis	(((((adult periodontitis[MeSH Terms]) OR (adult periodontitides[MeSH Terms])) OR (aggressive periodontitis[MeSH Terms])) OR (apical periodontitis[MeSH Terms])) OR (chronic periodontitis[MeSH Terms])) OR (“periodontiti*”[Title/Abstract])
Step 3: search for terms relating to gingivitis	((gingivitis [MeSH Terms]) OR (acute necrotizing ulcerative gingivitis[MeSH Terms])) OR (“gingivitis”[Title/Abstract])
**Step 4: Combine the results for steps 1-3 using an OR operation**	
Step 5: search for terms relating to clinical outcome	(((((((((((((gingival index*[MeSH Terms]) OR (plaque index*[MeSH Terms])) OR (attachment loss, periodontal[MeSH Terms])) OR (periodontal attachment loss[MeSH Terms])) OR (bleeding on probing, gingival[MeSH Terms])) OR (gingival bleeding on probing[MeSH Terms])) OR (“periodontal pocket”[MeSH Terms])) OR (“gingival inde*”[Title/Abstract])) OR (“plaque inde*”[Title/Abstract])) OR (“attachment loss”[Title/Abstract])) OR (“bleeding on probing”[Title/Abstract])) OR (“periodontal pocket”[Title/Abstract])) OR (“probing pocket depth”[Title/Abstract])) OR (“gingival bleeding”[Title/Abstract])
Step 6: search for terms relating to immunological outcome	((((((((((((((((((((((fluid, gingival crevicular[MeSH Terms]) OR (gingival crevicular fluid*[MeSH Terms])) OR (matrix metalloproteinase*[MeSH Terms])) OR (adaptive immunity[MeSH Terms])) OR ("immunity"[MeSH Terms])) OR (immune factors[MeSH Terms])))) OR (active immune response*[MeSH Terms])) OR (adaptive immune response*[MeSH Terms])) OR (acquired immunity[MeSH Terms])) OR (adoptive immunity[MeSH Terms])) OR (cytokines[MeSH Terms])) OR (gingival crevicular fluid*[Title/Abstract])) OR (matrix metalloprote*[Title/Abstract])) OR (immune system*[Title/Abstract])) OR (immune function*[Title/Abstract])) OR (immune response*[Title/Abstract])) OR (immunit*[Title/Abstract])) OR (inflam*atory respons*[Title/Abstract])) OR (cytokine*[Title/Abstract])) OR (inflam*atory factor*[Title/Abstract])) OR (immune factor*[Title/Abstract])
Step 7: search for terms relating to microbiological outcome	((((microbiom*[MeSH Terms]) OR (microbiota*[MeSH Terms])) OR (oral pathogen*[Title/Abstract])) OR (periodontopathogene*[Title/Abstract])) OR (periodontal pathogen*[Title/Abstract])
**Step 8: Combine the results for steps 5-7 using an OR operation**	
Step 9: search for terms relating to probiotics	(((probiotic[MeSH Terms]) OR (probiotics[MeSH Terms])) OR (fermented milk product[MeSH Terms])) OR (fermented milk products[MeSH Terms])) OR (milk product, fermented[MeSH Terms])) OR (milk products, fermented[MeSH Terms])) OR (product, fermented milk[MeSH Terms])) OR (probiotic*[Title/Abstract])) OR (probiotic food[Title/Abstract])) OR (fermented food[Title/Abstract])) OR (Lactobacillus rhamnosus[MeSH Terms])) OR (Lactobacillus reuteri[MeSH Terms])) OR (Lactobacillus[MeSH Terms])) OR (lactobacillus acidophilus[MeSH Terms])) OR (lactobacillus brevis[MeSH Terms])) OR (lactobacillus casei[MeSH Terms])) OR (bifidobacterium[MeSH Terms])) OR (Bifidobacterium

	bifidum[MeSH Terms])) OR (Bifidobacterium animalis[MeSH Terms])) OR (Bifidobacterium breve[MeSH Terms])) OR (Bifidobacterium adolescentis[MeSH Terms])) OR (Bifidobacterium longum[MeSH Terms])) OR (Bacillus[MeSH Terms])) OR (Bacillus clausii[MeSH Terms])) OR (Bacillus coagulans[MeSH Terms])) OR (Cultured Milk Products[MeSH Terms])) OR (yogurt[MeSH Terms])) OR (kefir[MeSH Terms])) OR (cheese[MeSH Terms])) OR ("yogurt"[Title/Abstract])) OR ("chees*"[Title/Abstract])) OR ("kefir"[Title/Abstract])) OR ("Lactobacillus reuteri"[Title/Abstract])) OR ("Lactobacillus"[Title/Abstract])) OR ("lactobacillus casei"[Title/Abstract])) OR ("Bifidobacterium"[Title/Abstract])) OR ("Bifidobacterium bifidum"[Title/Abstract])) OR ("Bifidobacterium animalis"[Title/Abstract])) OR ("Bifidobacterium breve"[Title/Abstract])) OR ("Bifidobacterium adolescentis"[Title/Abstract])) OR ("Bifidobacterium longum"[Title/Abstract])) OR ("Bacillus"[Title/Abstract])) OR ("Bacillus clausii"[Title/Abstract])) OR ("Bacillus coagulans"[Title/Abstract])) OR ("fermented vegetable*"[Title/Abstract])
**Step 10: Combine the results for step 4, 8 and step 9 using an AND operation**	
	((((((((periodontal diseases [MeSH Terms]) OR (periodontal disease [MeSH Terms])) OR ("periodontal disease*[Title/Abstract])) OR ("gingival disease*"[Title/Abstract])) OR ((((((adult periodontitis[MeSH Terms]) OR (adult periodontitides[MeSH Terms])) OR (aggressive periodontitis[MeSH Terms])) OR (apical periodontitis[MeSH Terms])) OR (chronic periodontitis[MeSH Terms])) OR (periodontiti*[Title/Abstract]))) OR (((gingivitis [MeSH Terms]) OR (acute necrotizing ulcerative gingivitis[MeSH Terms])) OR ("gingivitis"[Title/Abstract]))) AND (((((((((((((((((((((((((fluid, gingival crevicular[MeSH Terms]) OR (gingival crevicular fluid*[MeSH Terms])) OR (matrix metalloproteinase*[MeSH Terms])) OR (adaptive immunity[MeSH Terms])) OR ("immunity"[MeSH Terms])) OR (immune factors[MeSH Terms])))) OR (active immune response*[MeSH Terms])) OR (adaptive immune response*[MeSH Terms])) OR (acquired immunity[MeSH Terms])) OR (adoptive immunity[MeSH Terms])) OR (cytokines[MeSH Terms])) OR (gingival crevicular fluid*[Title/Abstract])) OR ("matrix metalloprote*"[Title/Abstract])) OR ("immune system*"[Title/Abstract])) OR ("immune function*"[Title/Abstract])) OR ("immune response*"[Title/Abstract])) OR ("immunit*"[Title/Abstract])) OR ("inflammatory respons*"[Title/Abstract])) OR ("cytokine*"[Title/Abstract])) OR ("inflammatory factor*"[Title/Abstract])) OR ("immune factor*"[Title/Abstract])) OR (((((microbiom*[MeSH Terms]) OR (microbiota*[MeSH Terms])) OR ("oral pathogen*"[Title/Abstract])) OR ("periodontopathogene*"[Title/Abstract])) OR ("periodontal pathogen*"[Title/Abstract]))) OR ((((((((((((((gingival index*[MeSH Terms]) OR (plaque index*[MeSH Terms])) OR (attachment loss, periodontal[MeSH Terms])) OR (periodontal attachment loss[MeSH Terms])) OR (bleeding on probing, gingival[MeSH Terms])) OR (gingival bleeding on probing[MeSH Terms])) OR (periodontal pocket[MeSH Terms])) OR ("gingival inde*"[Title/Abstract])) OR ("plaque inde*"[Title/Abstract])) OR ("attachment loss"[Title/Abstract])) OR ("bleeding on probing"[Title/Abstract])) OR ("periodontal pocket"[Title/Abstract])) OR ("probing pocket depth"[Title/Abstract])) OR ("gingival bleeding"[Title/Abstract])))) AND (((((((((((((((((((((((((((probiotic[MeSH Terms]) OR (probioti cs[MeSH Terms])) OR (fermented milk product[MeSH Terms])) OR (fermented milk products[MeSH Terms])) OR (milk product, fermented[MeSH Terms])) OR (milk products, fermented[MeSH Terms])) OR (product, fermented milk[MeSH Terms])) OR (probiotic*[Title/Abstract])) OR (probiotic food[Title/Abstract])) OR (fermented food[Title/Abstract])) OR (Lactobacillus rhamnosus[MeSH Terms])) OR (Lactobacillus reuteri[MeSH Terms])) OR (Lactobacillus[MeSH Terms])) OR (lactobacillus acidophilus[MeSH Terms])) OR (lactobacillus brevis[MeSH Terms])) OR (lactobacillus casei[MeSH Terms])) OR (bifidobacterium[MeSH Terms])) OR (Bifidobacterium bifidum[MeSH Terms])) OR (Bifidobacterium animalis[MeSH Terms])) OR (Bifidobacterium breve[MeSH Terms])) OR (Bifidobacterium adolescentis[MeSH Terms])) OR (Bifidobacterium

	longum[MeSH Terms])) OR (Bacillus[MeSH Terms])) OR (Bacillus clausii[MeSH Terms])) OR (Bacillus coagulans[MeSH Terms])) OR (Cultured Milk Products[MeSH Terms])) OR (yogurt[MeSH Terms])) OR (kefir[MeSH Terms])) OR (cheese[MeSH Terms])) OR ("yogurt"[Title/Abstract])) OR ("chees*"[Title/Abstract])) OR ("kefir"[Title/Abstract])) OR ("Lactobacillus reuteri"[Title/Abstract])) OR ("Lactobacillus"[Title/Abstract])) OR ("lactobacillus casei"[Title/Abstract])) OR ("Bifidobacterium"[Title/Abstract])) OR ("Bifidobacterium bifidum"[Title/Abstract])) OR ("Bifidobacterium animalis"[Title/Abstract])) OR ("Bifidobacterium breve"[Title/Abstract])) OR ("Bifidobacterium adolescentis"[Title/Abstract])) OR ("Bifidobacterium longum"[Title/Abstract])) OR ("Bacillus"[Title/Abstract])) OR ("Bacillus clausii"[Title/Abstract])) OR ("Bacillus coagulans"[Title/Abstract])) OR ("fermented vegetable*"[Title/Abstract]))

Supplemental Table S3: Risk of Bias within Studies

Study ID	Randomization process	Deviations from intended interventions	Missing outcome data	Measurement of the outcome	Selection of the reported result	Overall
Alkaya, 2017	+	+	+	+	+	+
Alshareef, 2020	−	?	−	−	+	−
Bazyar, 2020	+	+	+	+	+	+
Bollero, 2017	+	+	+	+	+	+
Boyeena, 2019	?	?	+	?	+	!
Chandra, 2016	+	?	+	+	+	!
Deshmukh, 2017	+	+	+	+	+	+
Dhaliwal, 2017	−	?	+	−	−	−
Duarte, 2019	?	?	+	?	−	−
Elsadek, 2020	?	?	+	?	+	!
Ercan, 2020	+	+	+	+	+	+
Grusovin, 2019	+	+	+	+	+	+
Hallström,2013	+	+	+	+	+	+
Ikram, 2018	+	+	+	+	+	+
Ikram, 2019	+	+	+	+	+	+
Ince, 2015	+	+	+	+	+	+
Iniesta, 2012	+	+	+	+	+	+
Invernici, 2018	+	+	+	+	+	+
Iwasakia, 2016	+	+	+	+	+	+
Jagadeesh, 2017	+	+	+	+	−	−
Jäsberg, 2018	+	+	+	+	+	+
Keller, 2018	+	+	+	+	+	+
Krasse, 2005	+	+	+	+	+	+
Kuka, 2019	+	+	+	+	+	+
Kuru, 2017	+	+	+	+	+	+
Laleman, 2015	+	+	+	+	+	+
Laleman, 2019	+	+	+	+	+	+
Lee, 2015	+	+	+	+	+	+
Mayanagi, 2009	+	+	+	+	+	+
Meenakshi, 2018	−	+	+	−	+	−
Mitic, 2017	−	?	−	−	+	−
Montero, 2017	+	+	+	+	+	+
Morales, 2016	+	+	+	+	+	+
Morales, 2017	?	?	+	+	+	!
Nadkerny, 2015	+	+	−	−	+	−
Nasry, 2018	+	+	?	+	+	!
Pelekos, 2019	+	+	+	+	?	!
Pelekos, 2020	+	+	+	+	+	+
Penala, 2015	+	+	+	+	+	+
Pudgar, 2020	+	+	+	+	+	+
Sabatini, 2017	?	+	+	+	−	−
Sajedinejad, 2018	+	+	+	+	?	!
Scariya, 2015	+	?	−	+	?	−
Schlagenhauf, 2020	+	+	+	+	+	+
Schlagenhauf,2018	+	+	+	+	+	+
Shah, 2013	−	+	+	−	+	−
Shah, 2017	−	+	+	−	+	−
Shetty, 2020	?	?	?	?	−	−
Shimauchi, 2008	+	+	+	+	+	+
Sinkiewicz, 2010	?	+	+	+	+	!
Sinulingga, 2020	+	?	+	?	+	!
Slawik, 2011	?	?	+	−	−	−
Staab, 2009	+	+	+	+	+	+
Suzuki, 2012	+	+	+	+	?	!
Tekce, 2015	+	+	+	+	+	+
Teughels, 2013	+	+	+	+	+	+
Theodoro, 2019	?	+	+	+	+	!
Tobita, 2018	?	+	+	+	+	!
Toiviainen, 2015	+	+	+	+	+	+
Twetman, 2009	+	+	+	+	+	+
Vicario, 2013	+	+	+	+	+	+
Vivekananda, 2010	+	+	+	+	+	+
Vohra, 2019	+	+	+	?	+	!
Yuki, 2019	+	+	+	+	+	+

+ Low risk
? Some concerns
− High risk

Supplemental Table S4. Overall Measure of Effects of Probiotic Supplementation on Clinical, Immunological and Microbiological Outcomes.

Overall Measure of Effects (Hedge's g SMD) of Probiotic on Clinical, Immunological and Microbiological parameters										
Parameters	Periodontal Disease Patients					Healthy Participants				
	SMD (95% CI)	Sample Size		I^2 (p-value)	Studies	SMD (95% CI)	Sample Size		I^2 (p-value)	Studies
		Probiotic	Control				Probiotic	Control		
**Clinical**										
PlI	0.557 (0.228, 0.885)	244	234	75.715 **(≤ 0.05)**	13	0.001 (-0.304, 0.306)	80	81	< 0.001 (> 0.05)	3
MPP	0.879 (0.308, 1.450)	226	229	87.544 **(≤ 0.05)**	11	N/A				
GI	0.920 (0.426, 1.414)	269	257	86.027 **(≤ 0.05)**	14	0.077	55	56	< 0.001 (> 0.05)	2
PPD	0.578 (0.365, 0.790)	489	483	62.840 **(≤ 0.05)**	27	N/A				
CAL	0.413 (0.262, 0.563)	339	334	< 0.001 **(≤ 0.05)**	19	N/A				
BOP	0.841 (0.479, 1.20)	377	377	86.750 **(≤ 0.05)**	20	N/A				
GCF	0.568 (0.235, 0.902)	69	69	< 0.001 **(≤ 0.05)**	4	-0.295 (-0.759, 0.168)	35	34	< 0.001 (> 0.05)	2
REC	0.048 (-0.234, 0.330)	92	94	< 0.001 (> 0.05)	5	N/A				
**Microbiological (Subgingival bacterial count)**										
P. gingivalis	0.402 (0.120, 0.685)	108	108	10.769 **(≤ 0.05)**	5	N/A				
F. nucleatum	0.392 (0.127, 0.658)	108	108	< 0.001 **(≤ 0.05)**	5	N/A				
T. forsythia	0.341 (0.050, 0.633)	89	88	< 0.001 **(≤ 0.05)**	4	N/A				
P. intermedia	0.205 (-0.103, 0.513)	78	79	< 0.001 (> 0.05)	4	N/A				
A. actinomycetemcomitans	0.200 (-0.082, 0.481)	94	94	< 0.001 (> 0.05)	5	N/A				
**Immunological (levels in the GCF)**										

MMP-8 (ng/mL)	0.819 (0.417, 1.221)	53	48	< 0.001 **(≤ 0.05)**	2	N/A
IL-6 (pg/mL)	0.361 (0.079, 0.644)	96	96	< 0.001 **(≤ 0.05)**	3	N/A
IL1β (pg/mL)	0.227 (-0.294, 0.749)	59	60	51.679 (> 0.05)	3	N/A
IL-8 (pg/mL)	0.181 (-0.272, 0.634)	45	45	< 0.001 (> 0.05)	2	N/A
IL-10 (pg/mL)	-0.029 (-0.481, 0.424)	36	36	< 0.001 (> 0.05)	2	N/A
TNF-α (pg/mL)	0.124 (-0.233, 0.482)	59	57	< 0.001 (> 0.05)	3	N/A

Note: Bold indicates statistically significant findings (p-value ≤ 0.05).

Abbreviations: SMD: Standardized Mean Difference; 95% CI: 95% Confidence interval; I^2: Measure of heterogeneity; N/A: Not applicable due to lack of studies; PlI: Plaque index; MPP: Mean plaque percentage; GI: Gingival index; PPD: Probing pocket depth; CAL: Clinical attachment level; BOP: Bleeding on probing; GCF: Gingival crevicular fluid; REC: Gingival recession; *P. gingivalis*: *Porphyromonas gingivalis*; *F. nucleatum*: *Fusobacterium nucleatum*; *T. forsythia*: *Tannerella forsythia*; *P. intermedia*: *Prevotella intermedia*; *A. actinomycetemcomitans*: *Aggregatibacter actinomycetemcomitans*; MMP-8: matrix metalloproteinase-8; IL: Interleukin.

Subgroup Analysis of the Associations between Probiotic Supplementation and Clinical Outcomes in Periodontal Disease Patients.

Results of the subgroup analysis by clinical parameters are presented in Table 2. Note, subgroup analysis was conducted solely on periodontal disease patients and not on healthy volunteers due to a lack of studies. The forest plots for the subgroup analysis of clinical parameters are depicted in Supplemental Figure S1.

1. Subgroup Analysis Examining the Effects of Probiotic Supplementation on Plaque Index (P1I)

1.A. Type of periodontal disease

Subgroup analysis based on the type of periodontal disease (gingivitis or periodontitis) indicated a statistically significant reduction in PlI with probiotic supplementation in patients with periodontitis (SMD=0.736, 95% CI: 0.267, 1.206, I^2=71.842, p-value ≤ 0.05, n=8 studies).

1.B. Type of probiotic strain

Subgroup analysis by type of probiotic strain (*Lactobacillus*, mixed or other) showed statistically significant reductions in PlI of periodontal disease patients who received only *lactobacillus* species containing probiotic supplementation (SMD=0.639, 95% CI: 0.169,1.110, I^2=75.533, p-value ≤ 0.05, n=9 studies) compared to controls.

1. C. Type of *lactobacillus* species

Subgroup analysis by type of lactobacillus species (*L. reuteri* or other *lactobacillus*) indicated a statistically significant reduction in PlI among periodontal disease patients receiving only *lactobacillus reuteri* containing probiotic supplementation (SMD=0.707, 95% CI: 0.034, 1.381, I^2=80.976, p-value ≤ 0.05, n=5 studies) compared to controls.

1. D. Treatment duration

Subgroup analysis by treatment duration indicated a statistically significant reduction in PlI among periodontal disease patients receiving probiotic supplementation for up to one month (SMD=0.615, 95% CI: 0.146, 1.084, I^2=75.448, p-value ≤ 0.05, n=9 studies) compared to controls.

1. E. Mode of probiotic delivery

The subgroup analysis examining the effect of probiotic supplementation on periodontal disease patients by mode of probiotic delivery found statistically significant reductions in the PlI due the "oral and ingestion" mode of delivery of probiotic supplementation (SMD=0.495, 95% CI: 0.061, 0.930, I^2=71.689, p-value ≤ 0.05, n=8 studies) compared to controls.

1. F. Oral hygiene instructions

Subgroup analysis found statistically significant reductions in PlI for those who received oral hygiene instructions (SMD=0.622, 95% CI: 0.204, 1.040, I^2=66.923, p-value ≤ 0.05, n=7 studies), compared to controls.

2. Subgroup Analysis Examining the Effects of Probiotic Supplementation on Mean Plaque Percentage (MPP)

2. A. Type of periodontal disease

Subgroup analysis indicated a statistically significant reduction in MPP with probiotic supplementation in patients with periodontitis (SMD=0.681, 95% CI: 0.072, 1.290, I^2=82.212, p-value ≤ 0.05, n=8 studies) compared to controls.

2. B. Type of probiotic strain

Subgroup analysis showed a statistically significant reduction in MPP in periodontal disease patients receiving only *lactobacillus* species containing probiotic supplementation (SMD=1.037, 95% CI: 0.391, 1.683, I^2=88.278, p-value ≤ 0.05, n=9 studies) compared to controls.

2. C. Type of *lactobacillus* species

Periodontal disease patients receiving only *L. reuteri* containing probiotic supplementation had a statistically significant decrease in their MPP (SMD=1.458, 95% CI: 0.724, 2.191, I^2=86.723, p-value ≤ 0.05, n=6 studies) compared to controls.

2. D. Treatment duration

Subgroup analysis found statistically significant decreases in the MPP for probiotic supplementation lasting for up to one month (SMD=0.937, 95% CI: 0.076, 1.798, I^2=90.960, p-value ≤ 0.05, n=7 studies), and more than two months (SMD=0.460, 95% CI: 0.008, 0.912, I^2=21.718, p-value ≤ 0.05, n=3 studies) compared to controls.

2. E. Mode of probiotic delivery

Probiotic supplementation resulted in a statistically significant decrease in the MPP with the oral and ingestion (SMD=0.942, 95% CI: 0.159, 1.725, I^2=87.844, p-value ≤ 0.05, n=6 studies) mode of delivery compared to controls.

2. F. Oral hygiene instructions

Subgroup analysis found statistically significant reductions in the MPP with probiotic supplementation and oral hygiene instructions (SMD=0.880, 95% CI: 0.197, 1.564, I^2=88.210, p-value ≤ 0.05, n=9 studies) compared to controls.

3. Subgroup Analysis Examining the Effects of Probiotic Supplementation on Gingival Index (GI)

3. A. Type of periodontal disease

There was a statistically significant reduction in the GI with probiotic supplementation among periodontitis patients compared to controls (SMD=1.069, 95% CI: 0.298, 1.841, I^2=86.299, p-value ≤ 0.05, n=7 studies).

3. B. Type of probiotic strain

Subgroup analysis by probiotic strain found a statistically significant reduction in the gingival index with probiotic supplementation for periodontal disease patients who received only *lactobacillus* species containing probiotics compared to controls (SMD=1.236, 95% CI: 0.574, 1.897, I^2=87.366, p-value ≤ 0.05, n=10 studies).

3. C. Type of *lactobacillus* species

When examining the effect of probiotic supplementation among periodontal disease patients stratified by type of *lactobacillus* species, both *L. reuteri* containing probiotics (SMD=1.621, 95% CI: 0.648, 2.595, I^2=89.871, p-value ≤ 0.05, n=5 studies) and other *lactobacillus* species-containing probiotics (SMD=0.817, 95% CI: 0.018, 1.616, I^2=79.137, p-value ≤ 0.05, n=5 studies) had statistically significant reductions in the GI compared to controls.

3. D. Treatment duration

There was a statistically significant reduction of GI in periodontal disease patients with probiotic supplementation treatment durations lasting up to one month (SMD=0.949, 95% CI: 0.270, 1.628, I^2=85.079, p-value ≤ 0.05, n=8 studies) compared to controls.

3. E. Mode of probiotic delivery

Subgroup analysis found that the probiotic supplementation group with the oral and ingestion mode of delivery showed a statistically significant reduction in GI compared to controls (SMD=1.051, 95% CI: 0.306, 1.797, I^2=89.846, p-value ≤ 0.05, n=8 studies).

3. F. Oral hygiene instructions

Subgroup analysis by oral hygiene instructions found a statistically significant decrease in GI in the probiotic supplementation group compared to the controls among periodontal disease patients who received oral hygiene instructions (SMD=1.051, 95% CI: 0.327, 1.775, I^2=86.466, p-value ≤ 0.05, n=4 studies) and who did not receive oral hygiene instructions (SMD=1.344, 95% CI: 0.261, 2.427, I^2=89.898, p-value ≤ 0.05, n=4 studies) compared to their respective controls.

4. Subgroup Analysis Examining the Effects of Probiotic Supplementation on Probing Pocket Depth (PPD)

4. A. Type of periodontal disease

Subgroup analysis examining type of periodontal disease, showed statistically significant decreases in the PPD among periodontitis patients with probiotic supplementation (SMD=0.578, 95% CI: 0.355, 0.801, I^2=62.716, p-value ≤ 0.05, n=25 studies) when compared to controls.

4. B. Type of probiotic strain

The subgroup analysis examining the effect of type of probiotic strain on periodontal disease patients found statistically significant reductions in the probiotic supplementation groups on the PPD with *lactobacillus* (SMD=0.674; 95% CI: 0.386, 0.962; I^2=69.524, p-value ≤ 0.05, n=19 studies) and "mixed" (SMD=0.387, 95% CI: 0.045, 0.729, I^2<0.001, p-value ≤ 0.05, n=4 studies) probiotic formulations compared to controls.

4. C. Type of *lactobacillus* species

The subgroup analysis examining the effect of the type of *lactobacillus* probiotic strain on periodontal disease patients found statistically significant reductions in the PPD with probiotic supplementation compared to controls when using probiotic containing *lactobacillus* irrespective of *L. reuteri* (SMD=677; 95% CI: 0.315, 1.040; I^2=74.541, p-value ≤ 0.05, n=13 studies) or other *lactobacillus* strains (SMD=0.657; 95% CI: 0.169, 1.144; I^2=56.911, p-value ≤ 0.05, n=6 studies).

4. D. Treatment duration

Subgroup analysis by treatment duration indicated statistically significant decreases in PPD with probiotic supplementation treatments lasting up to one month (SMD=0.737; 95% CI: 0.430, 1.044; I^2=66.736, p-value ≤ 0.05, n=15 studies) and more than two months (SMD=0.326; 95% CI: 0.015, 0.636; I^2=43.080, p-value ≤ 0.05, n=9 studies) compared to controls.

4. E. Mode of probiotic delivery

When examining mode of probiotic delivery, ingestion (SMD=0.514, 95% CI: 0.106, 0.922, I^2=47.870, p-value ≤ 0.05, n=6 studies) and, oral and ingestion (SMD=0.525; 95% CI: 0.251, 0.800; I^2=66.577, p-value ≤ 0.05, n=17 studies) use of probiotic led to statistically significant reductions of the PPD in patients with periodontal disease compared to controls.

4. F. Oral hygiene instructions

In subgroup analysis, the pooled SMD of the studies with (SMD=0.592; 95% CI: 0.343, 0.841; I^2=63.935, p-value ≤ 0.05, n=21 studies) and without (SMD=0.974; 95% CI: 0.307, 1.641; I^2=66.484, p-value ≤ 0.05, n=3 studies) oral hygiene instructions showed statistically significant decreases in PPD with probiotic supplementation compared to controls.

4. G. Disease severity

Subgroup analysis examining disease severity in periodontal disease patients showed statistically significant decreases in the PPD with probiotic supplementation for both moderate (SMD=0.499, 95% CI: 0.043, 0.955, I^2=66.202, p-value ≤ 0.05, n=6 studies) and deep

periodontal pockets (SMD=0.735, 95% CI: 0.209, 1.261, I^2=73.585 p-value ≤ 0.05, n=6 studies) compared to controls.

4. *H.* Comparison of probiotic and antibiotic use on probing pocket depth

In addition to using placebo/without probiotic control groups, meta-analysis was conducted to examine the effect of probiotic supplementation compared to antibiotic use on PPD. There was no statistically significant difference in the PPD when comparing periodontal disease patients receiving probiotic supplementation with those receiving antibiotics (SMD=0.630, 95% CI: -0.096, 1.356, I^2=69.610, p-value ≤ 0.05, n=4 studies). We did not find evidence of publication bias or small study effects upon visual inspection of the funnel plot and with Egger's regression test (p-value > 0.05).

5. Subgroup Analysis Examining the Effects of Probiotic Supplementation on Clinical Attachment Level (CAL)

5. A. Type of probiotic strain

The subgroup analysis examining the effect of type of probiotic strain on periodontitis patients found statistically significant CAL gain due to *lactobacillus* (SMD=0.417, 95% CI: 0.225, 0.609, I^2=8.881, p-value ≤ 0.05, n=14 studies) and other (SMD=0.415, 95% CI: 0.076, 0.755, I^2=7.610, p-value ≤ 0.05, n=3 studies) probiotic formulations compared to controls (with placebo or without probiotic).

5. B. Type of *lactobacillus* species

The subgroup analysis examining the effect of the type of *lactobacillus* probiotic strain on periodontitis patients found statistically significant CAL gain when using probiotic supplementation containing *lactobacillus reuteri* (SMD=0.416, 95% CI: 0.201, 0.631, I^2=12.027, p-value ≤ 0.05, n=11 studies) compared to controls.

5. C. Treatment duration

In subgroup analysis, irrespective of treatment duration, there was a statistically significant CAL gain in patients with periodontitis with probiotic supplementation for up to one month (SMD=0.388, 95% CI: 0.185, 0.592, I^2 < 0.001, p-value ≤ 0.05, n=10 studies), more than one month to two months (SMD=0.789, 95% CI: 0.236, 1.343, I^2=34.507, p-value ≤ 0.05, n=2 studies) and more than two months (SMD=0.330, 95% CI: 0.071, 0.588, I^2 < 0.001, p-value ≤ 0.05, n=7 studies) compared to controls.

5. D. Mode of probiotic delivery

Subgroup analysis based on mode of probiotic delivery found ingestion (SMD=0.464, 95% CI: 0.116, 0.812, I^2=0.276, p-value ≤ 0.05, n= 4 studies) and, oral and ingestion (SMD=0.339, 95% CI: 0.159, 0.520, I^2 < 0.001, p-value ≤ 0.05, n=13 studies) use of probiotic supplementation led to statistically significant CAL gain in patients with periodontitis compared to controls.

5. E. Oral hygiene instructions

Probiotic supplementation led to statistically significant CAL gain in periodontitis patients regardless of the presence (SMD=0.351, 95% CI: 0.178, 0.523, I^2 < 0.001, p-value ≤ 0.05, n=14 studies) or absence (SMD=0.835, 95% CI: 0.437, 1.233, I^2 < 0.001, p-value ≤ 0.05, n=3 studies) of oral hygiene instructions.

5. F. Disease severity

The subgroup analysis examining disease severity indicated a statistically significant CAL gain in periodontitis patients with probiotic supplementation in both moderate (SMD=0.422, 95% CI: 0.137, 0.706, I^2 < 0.001, p-value ≤ 0.05, n=5 studies) and deep (SMD=0.373,

95% CI: 0.088, 0.657, $I^2 < 0.001$, p-value ≤ 0.05, n=5 studies) periodontal pockets compared to controls.

5. G. Comparison of probiotic and antibiotic use on clinical attachment level

In addition to using placebo/without probiotic control groups, meta-analysis was conducted to examine the effects of probiotic supplementation compared to antibiotic use on CAL. The pooled meta-analysis found no statistically significant difference in CAL when comparing probiotic supplementation and antibiotic control groups in individuals with periodontal disease (SMD=0.636, 95% CI: -0.290, 1.562, I^2=76.848, p-value ≤ 0.05, n=3 studies). We did not find evidence of publication bias or small study effects upon visual inspection of the funnel plot and with Egger's regression test (p-value > 0.05).

6. Subgroup Analysis Examining the Effects of Probiotic Supplementation on Bleeding on Probing (BOP)

6. A. Type of periodontal disease

Subgroup analysis based on the type of periodontal disease indicated statistically significant decreases in BOP in patients with periodontitis with probiotic supplementation compared to controls (SMD=0.749, 95% CI: 0.404, 1.094, I^2=72.526, p-value ≤ 0.05, n=15 studies).

6. B. Type of probiotic strain

The subgroup analysis examining the effect of the type of probiotic strain on periodontal disease patients found statistically significant reductions in BOP with probiotic supplementation due to *lactobacillus* (SMD=0.878, 95% CI: 0.442, 1.313, I^2=85.057, p-value ≤ 0.05, n=17 studies) probiotic formulations compared to controls.

6. C. Type of *lactobacillus* species

The subgroup analysis examining the effect of the type of *lactobacillus* probiotic strain on periodontal disease patients found statistically significant reductions in BOP with probiotic supplementation containing *L. reuteri* (SMD=1.054, 95% CI: 0.485, 1.622, I^2=86.818, p-value ≤ 0.05, n=11 studies) compared to controls.

6. D. Treatment duration

Subgroup analysis found probiotic supplementation resulted in statistically significant decreases in BOP in periodontal disease patients with treatment durations lasting for up to one month (SMD=1.024, 95% CI: 0.454-1.595, I^2=88.021, p-value ≤ 0.05, n=12 studies) and more than two months (SMD=0.402, 95% CI: 0.020, 0.785, I^2=55.314, p-value ≤ 0.05, n=7 studies) compared to controls. There was insufficient data for probiotic treatment durations of more than one month to two months to complete a meta-analysis.

6. E. Mode of probiotic delivery

Probiotic supplementation resulted in a statistically significant decrease in BOP with the oral and ingestion mode of delivery compared to controls in patients with periodontal disease (SMD=0.616, 95% CI=0.296, 0.936, I^2=60.399, p-value ≤ 0.05, n=11 studies).

6. F. Oral hygiene instructions

Probiotic supplementation with oral hygiene instructions resulted in a statistically significant decrease in BOP compared to controls in patients with periodontal disease (SMD= 0.966, 95% CI=0.478, 1.454, I^2=86.250, p-value ≤ 0.05, n=15 studies) compared to controls. There were insufficient studies to assess the effect of probiotic supplementation in the absence of oral hygiene instructions.

7. Subgroup Analysis Examining the Effects of Probiotic Supplementation on Gingival Crevicular Fluid (GCF)

Subgroup analysis based on the type of periodontal disease indicated statistically significant decreases in the GCF volume with probiotic supplementation compared to controls in both patients with periodontitis (SMD=0.507 95% CI: 0.027, 0.986, $I^2<0.001$, p-value ≤ 0.05, n=2 studies) and gingivitis (SMD= 0.626, 95% CI = 0.162, 1.091, $I^2 < 0.001$, p-value ≤ 0.05, n=2 studies). Additional subgroup analysis was not possible due to a lack of studies.

8. Subgroup Analysis Examining the Effects of Probiotic Supplementation on Gingival Recession (REC)

Subgroup analysis by treatment duration and type of probiotic strain was not statistically significant. A lack of studies prevented additional subgroup analysis for REC regarding mode of probiotic delivery and oral hygiene instructions.

Supplemental Figure S1: Subgroup Analysis Forest Plots Examining the Effects of Probiotic Supplementation on Clinical Outcomes

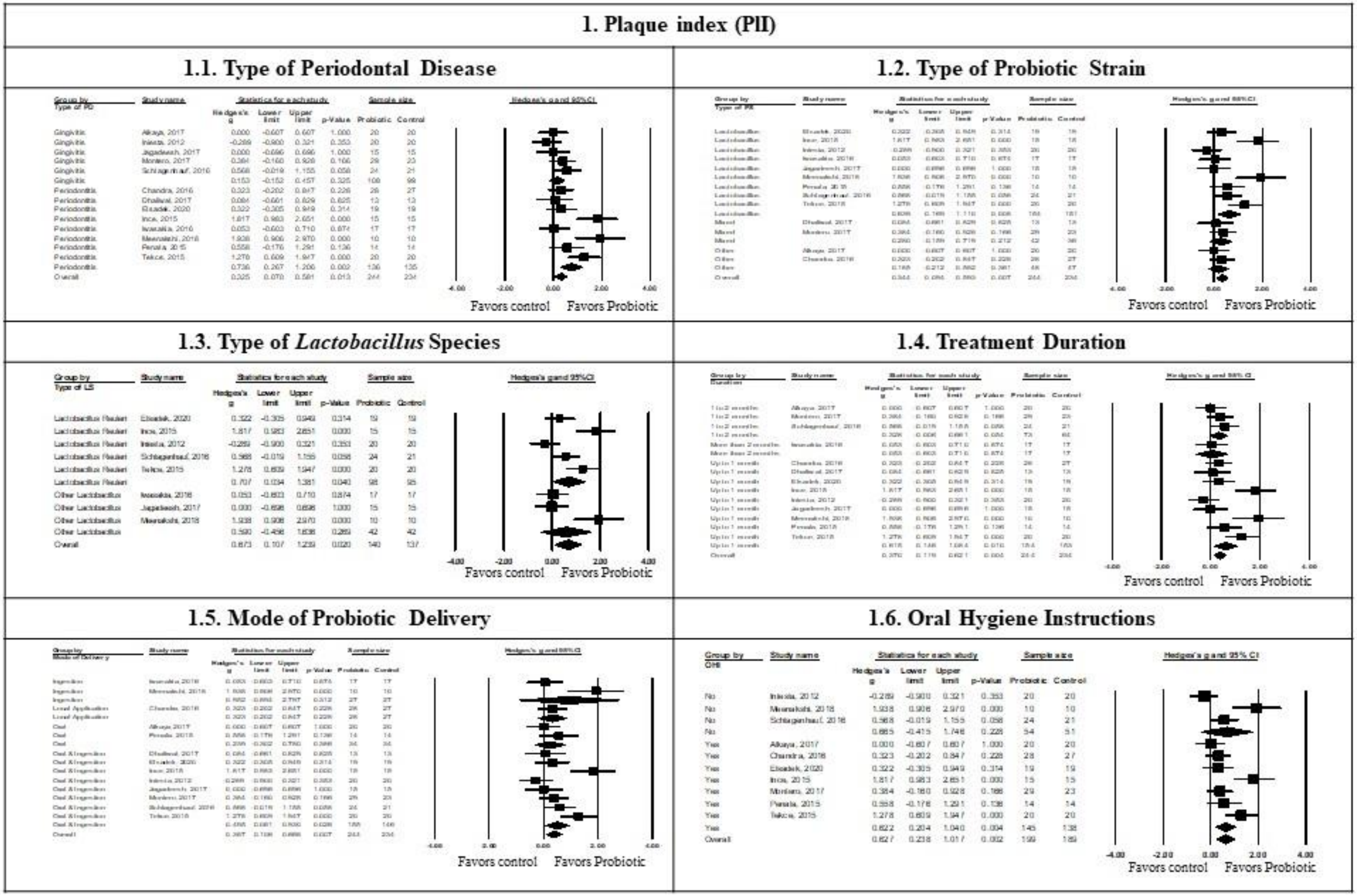

2. Mean Plaque Percentage Change (MPP)

2.1. Type of Periodontal Disease

Group by Type of PD	Study name	Hedges's g	Lower limit	Upper limit	p-Value	Probiotic	Control
		Statistics for each study				Sample size	
Gingivitis	Keller, 2018	0.165	-0.398	0.729	0.565	23	24
Gingivitis	Sabatini, 2017	2.394	1.823	2.965	0.000	40	40
Gingivitis		1.279	-0.905	3.463	0.251	63	64
Periodontitis	Alshareef, 2020	0.112	-0.728	0.952	0.794	10	10
Periodontitis	Ikram, 2019	0.972	0.209	1.734	0.013	14	14
Periodontitis	Invernici, 2018	0.199	-0.396	0.794	0.513	21	21
Periodontitis	Laleman, 2019	0.198	-0.411	0.807	0.523	20	20
Periodontitis	Morales, 2016	0.331	-0.393	1.056	0.370	14	14
Periodontitis	Sajedinejad, 2017	0.067	-0.773	0.907	0.875	10	10
Periodontitis	Vicario, 2013	1.101	0.172	2.030	0.020	10	9
Periodontitis	Vohra, 2019	2.442	1.794	3.089	0.000	31	32
Periodontitis		0.681	0.072	1.290	0.028	130	130
Overall		0.724	0.137	1.311	0.016	193	194

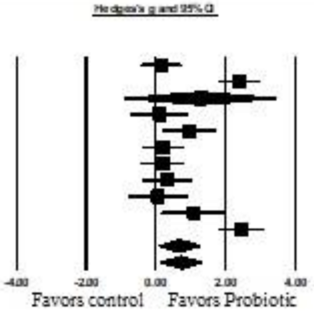

2.2. Type of Probiotic Strain

Group by Type of PS	Study name	Hedges's g	Lower limit	Upper limit	p-Value	Probiotic	Control
		Statistics for each study				Sample size	
Lactobacillus	Ikram, 2019	0.972	0.209	1.734	0.013	14	14
Lactobacillus	Keller, 2018	0.165	-0.398	0.729	0.565	23	24
Lactobacillus	Laleman, 2019	0.198	-0.411	0.807	0.523	20	20
Lactobacillus	Morales, 2016	0.331	-0.393	1.056	0.370	14	14
Lactobacillus	Sabatini, 2017	2.394	1.823	2.965	0.000	40	40
Lactobacillus	Sajedinejad, 2017	0.067	-0.773	0.907	0.875	10	10
Lactobacillus	Schlagenhauf, 2020	1.560	1.022	2.099	0.000	33	35
Lactobacillus	Vicario, 2013	1.101	0.172	2.030	0.020	10	9
Lactobacillus	Vohra, 2019	2.442	1.794	3.089	0.000	31	32
Lactobacillus		1.037	0.391	1.683	0.002	195	198
Mixed	Alshareef, 2020	0.112	-0.728	0.952	0.794	10	10
Mixed		0.112	-0.728	0.952	0.794	10	10
Other	Invernici, 2018	0.199	-0.396	0.794	0.513	21	21
Other		0.199	-0.396	0.794	0.513	21	21
Overall		0.483	0.094	0.871	0.015	226	229

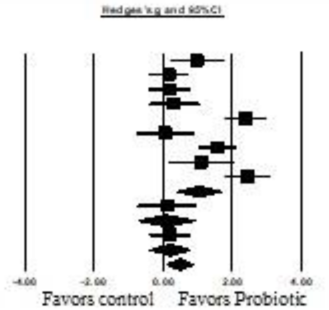

2.3. Type of *Lactobacillus* Species

Group by Type of LS	Study name	Hedges's g	Lower limit	Upper limit	p-Value	Probiotic	Control
		Statistics for each study				Sample size	
Lactobacillus Reuteri	Ikram, 2019	0.972	0.209	1.734	0.013	14	14
Lactobacillus Reuteri	Laleman, 2019	0.198	-0.411	0.807	0.523	20	20
Lactobacillus Reuteri	Sabatini, 2017	2.394	1.823	2.965	0.000	40	40
Lactobacillus Reuteri	Schlagenhauf, 2020	1.560	1.022	2.099	0.000	33	35
Lactobacillus Reuteri	Vicario, 2013	1.101	0.172	2.030	0.020	10	9
Lactobacillus Reuteri	Vohra, 2019	2.442	1.794	3.089	0.000	31	32
Lactobacillus Reuteri		1.458	0.724	2.191	0.000	148	150
Other	Keller, 2018	0.165	-0.398	0.729	0.565	23	24
Other	Morales, 2016	0.331	-0.393	1.056	0.370	14	14
Other	Sajedinejad, 2017	0.067	-0.773	0.907	0.875	10	10
Other		0.193	-0.200	0.586	0.337	47	48
Overall		0.475	0.128	0.821	0.007	195	198

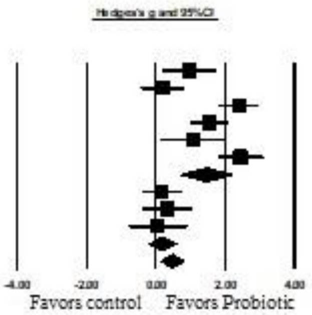

2.4. Treatment Duration

Group by Treatment Duration	Study name	Hedges's g	Lower limit	Upper limit	p-Value	Probiotic	Control
		Statistics for each study				Sample size	
1 to 2 months	Schlagenhauf, 2020	1.560	1.022	2.099	0.000	33	35
1 to 2 months		1.560	1.022	2.099	0.000	33	35
More than 2 months	Ikram, 2019	0.972	0.209	1.734	0.013	14	14
More than 2 months	Laleman, 2019	0.198	-0.411	0.807	0.523	20	20
More than 2 months	Morales, 2016	0.331	-0.393	1.056	0.370	14	14
More than 2 months		0.460	0.008	0.912	0.046	48	48
Up to 1 month	Alshareef, 2020	0.112	-0.728	0.952	0.794	10	10
Up to 1 month	Invernici, 2018	0.199	-0.396	0.794	0.513	21	21
Up to 1 month	Keller, 2018	0.165	-0.398	0.729	0.565	23	24
Up to 1 month	Sabatini, 2017	2.394	1.823	2.965	0.000	40	40
Up to 1 month	Sajedinejad, 2017	0.067	-0.773	0.907	0.875	10	10
Up to 1 month	Vicario, 2013	1.101	0.172	2.030	0.020	10	9
Up to 1 month	Vohra, 2019	2.442	1.794	3.089	0.000	31	32
Up to 1 month		0.937	0.076	1.798	0.033	145	146
Overall		0.918	0.597	1.239	0.000	226	229

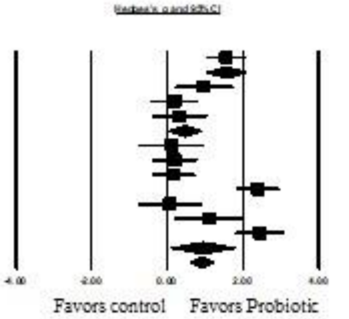

2.5. Mode of Probiotic Delivery

Group by Mode of Delivery	Study name	Hedges's g	Lower limit	Upper limit	p-Value	Probiotic	Control
		Statistics for each study				Sample size	
Ingestion	Keller, 2018	0.165	-0.398	0.729	0.565	23	24
Ingestion	Morales, 2016	0.331	-0.393	1.056	0.370	14	14
Ingestion	Sabatini, 2017	2.394	1.823	2.965	0.000	40	40
Ingestion		0.969	-0.506	2.445	0.198	77	78
Oral	Ikram, 2019	0.972	0.209	1.734	0.013	14	14
Oral	Sajedinejad, 2017	0.067	-0.773	0.907	0.875	10	10
Oral		0.537	-0.348	1.423	0.235	24	24
Oral & Ingestion	Alshareef, 2020	0.112	-0.728	0.952	0.794	10	10
Oral & Ingestion	Invernici, 2018	0.199	-0.396	0.794	0.513	21	21
Oral & Ingestion	Laleman, 2019	0.198	-0.411	0.807	0.523	20	20
Oral & Ingestion	Schlagenhauf, 2020	1.560	1.022	2.099	0.000	33	35
Oral & Ingestion	Vicario, 2013	1.101	0.172	2.030	0.020	10	9
Oral & Ingestion	Vohra, 2019	2.442	1.794	3.089	0.000	31	32
Oral & Ingestion		0.942	0.158	1.725	0.018	125	127
Overall		0.793	0.247	1.338	0.004	226	229

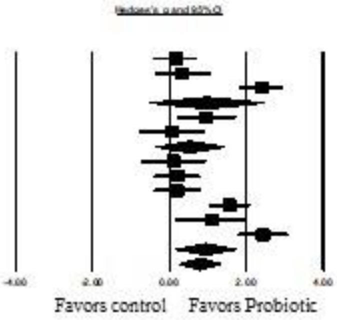

2.6. Oral Hygiene Instructions

Group by OHI	Study name	Hedges's g	Lower limit	Upper limit	p-Value	Probiotic	Control
		Statistics for each study				Sample size	
No	Keller, 2018	0.165	-0.398	0.729	0.565	23	24
No	Schlagenhauf, 2020	1.560	1.022	2.099	0.000	33	35
No		0.865	-0.502	2.232	0.215	56	59
Yes	Alshareef, 2020	0.112	-0.728	0.952	0.794	10	10
Yes	Ikram, 2019	0.972	0.209	1.734	0.013	14	14
Yes	Invernici, 2018	0.199	-0.396	0.794	0.513	21	21
Yes	Laleman, 2019	0.198	-0.411	0.807	0.523	20	20
Yes	Morales, 2016	0.331	-0.393	1.056	0.370	14	14
Yes	Sabatini, 2017	2.394	1.823	2.965	0.000	40	40
Yes	Sajedinejad, 2017	0.067	-0.773	0.907	0.875	10	10
Yes	Vicario, 2013	1.101	0.172	2.030	0.020	10	9
Yes	Vohra, 2019	2.442	1.794	3.089	0.000	31	32
Yes		0.880	0.197	1.564	0.012	170	170
Overall		0.877	0.266	1.488	0.005	226	229

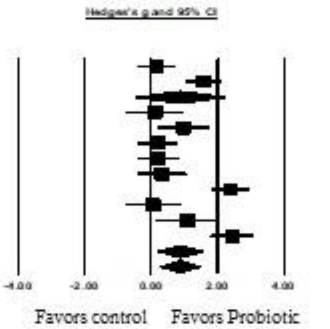

3. Gingival Index (GI)

3.1. Type of Periodontal Disease

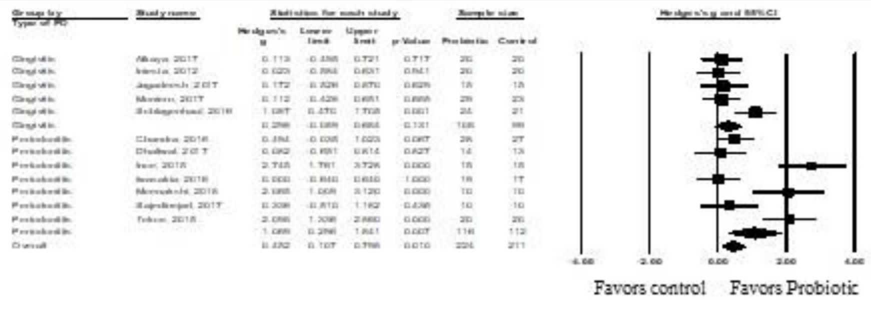

3.2. Type of Probiotic Strain

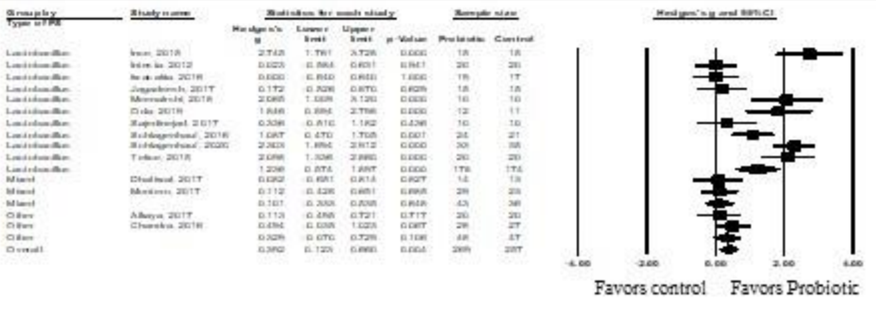

3.3. Type of *Lactobacillus* Species

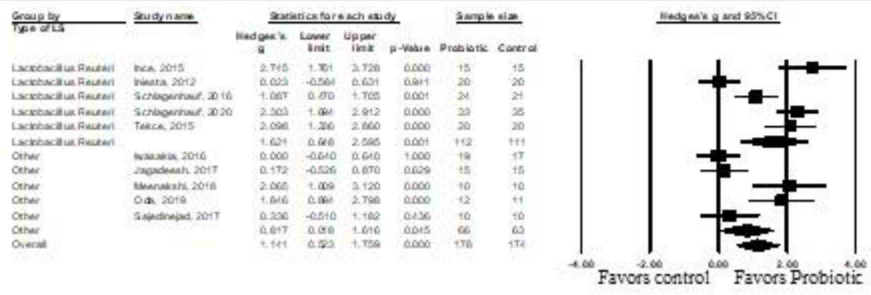

3.4. Treatment Duration

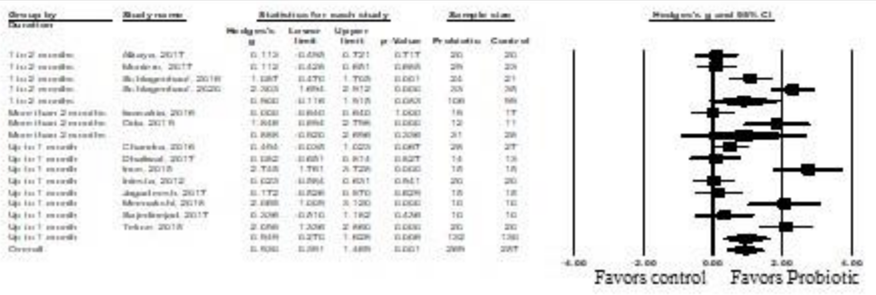

3.5. Mode of Probiotic Delivery

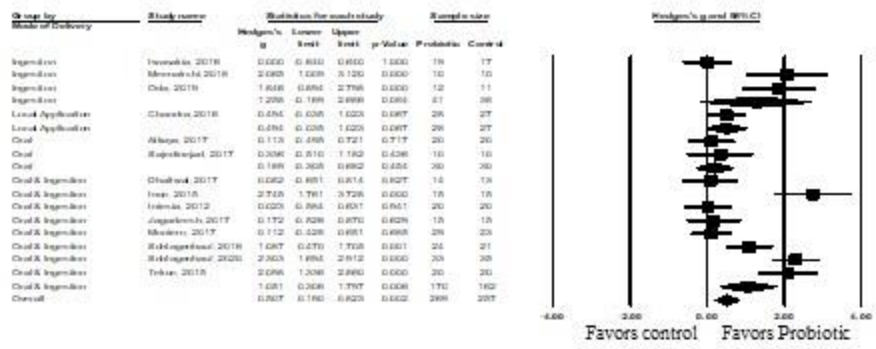

3.6. Oral Hygiene Instructions

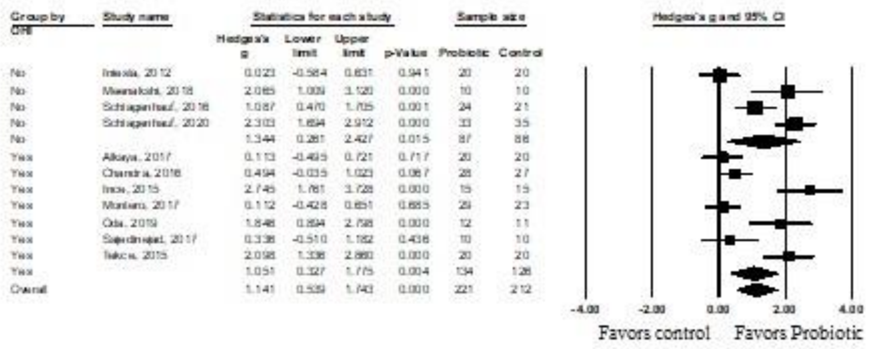

4. Probing Pocket Depth (PPD)

4.1. Type of Periodontal Disease

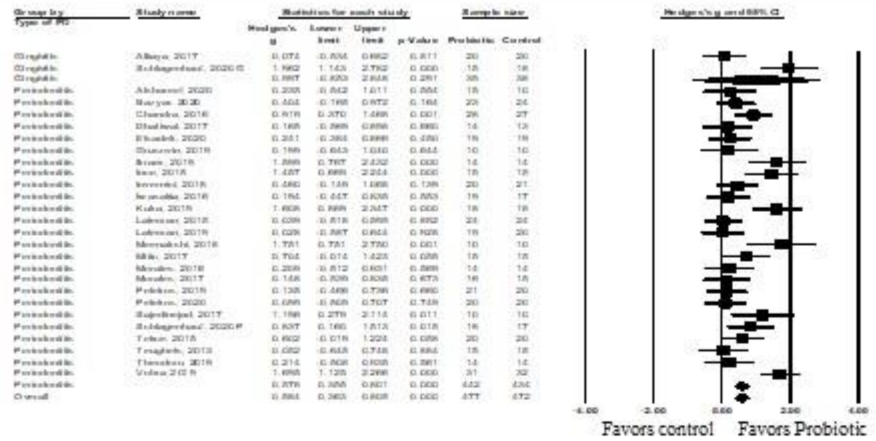

4.2. Type of Probiotic Strain

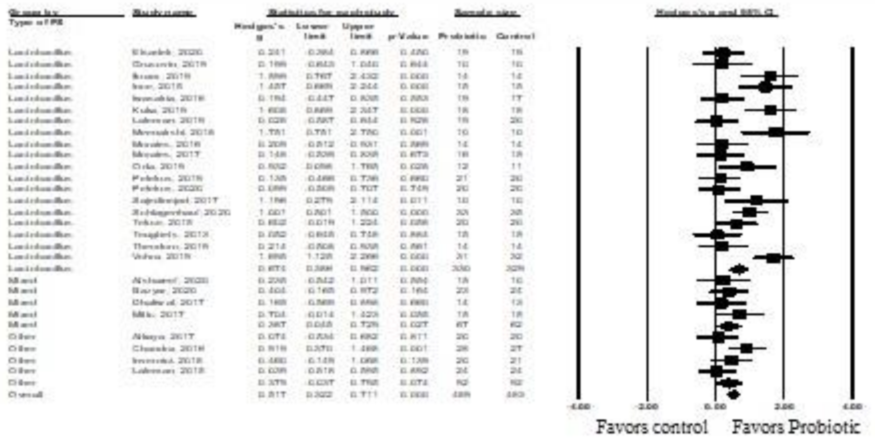

4.3. Type of *Lactobacillus* Species

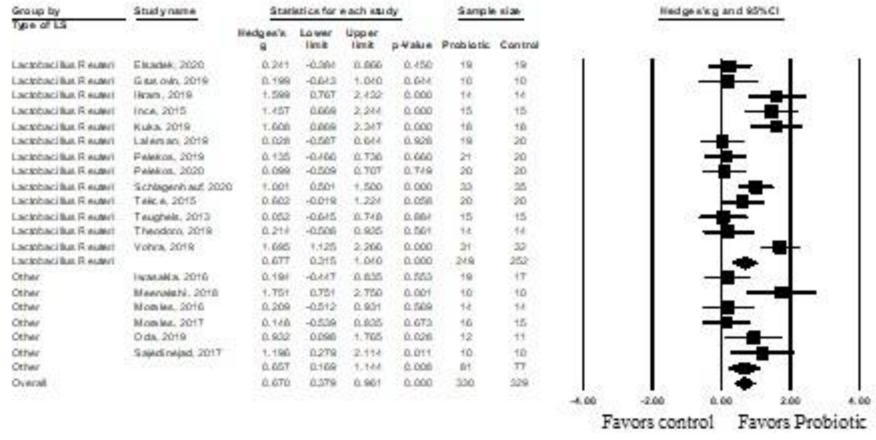

4.4. Treatment Duration

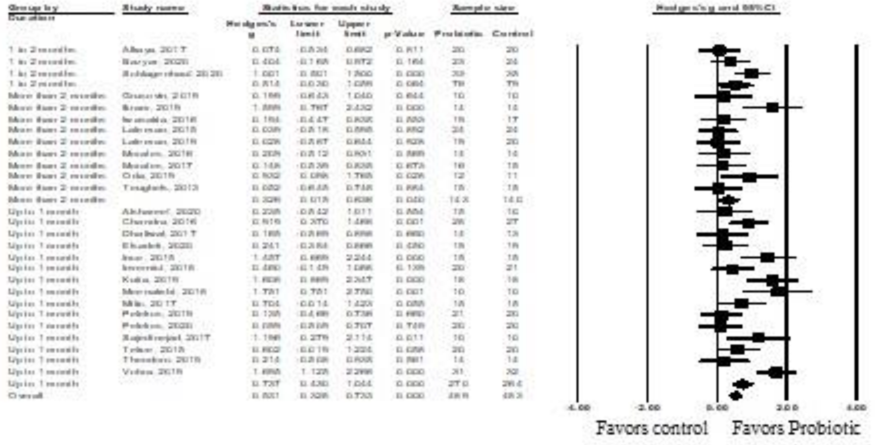

4.5. Mode of Probiotic Delivery

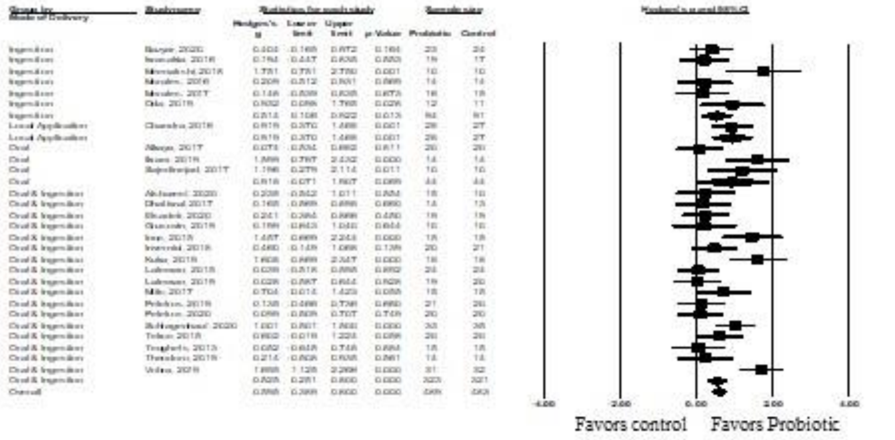

4.6. Oral Hygiene Instructions

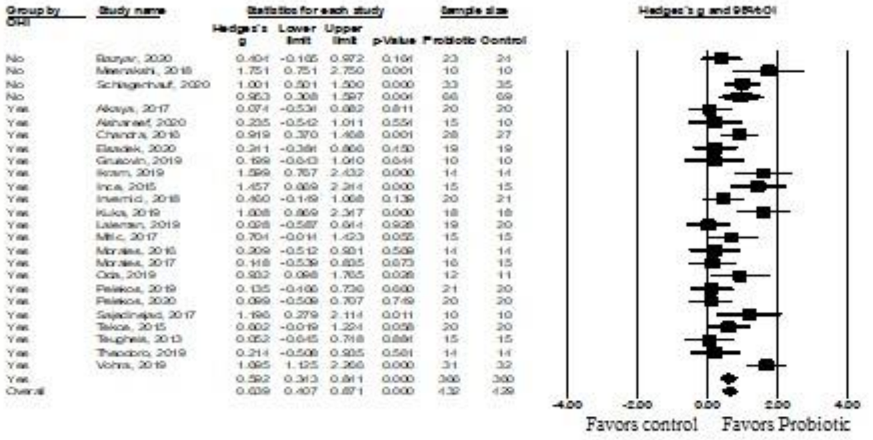

4.7. Disease Severity

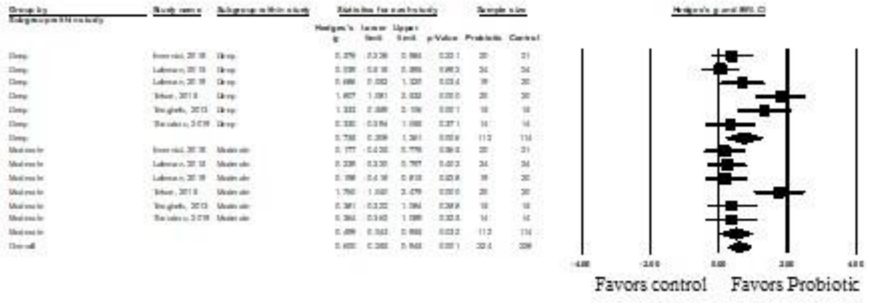

4.8. Comparison between Probiotics to Antibiotics

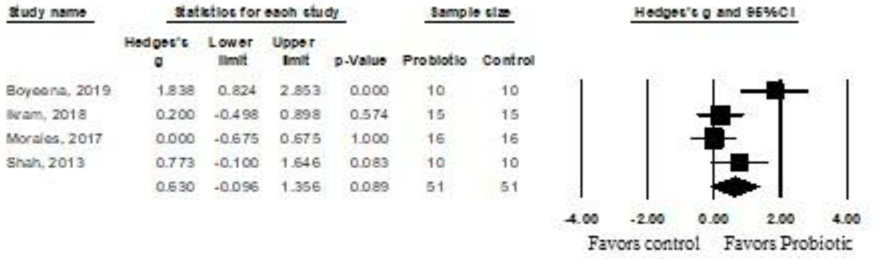

5. Clinical Attachment Level (CAL)

5.1. Type of Probiotic Strain

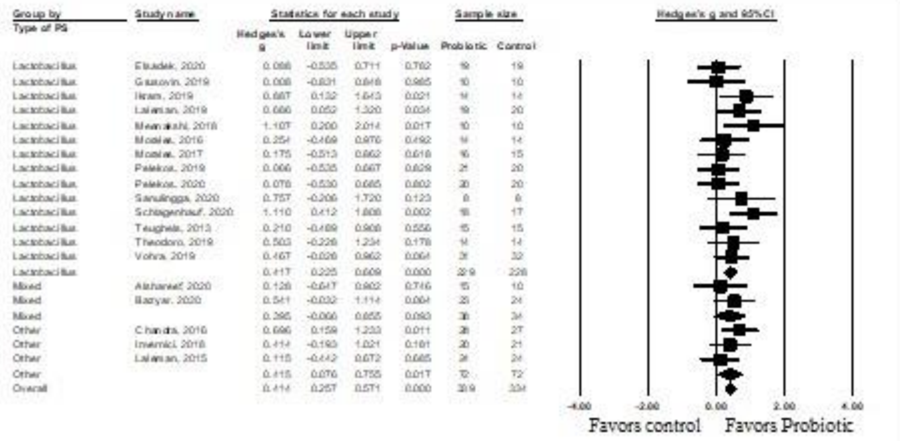

5.2. Type of *Lactobacillus* Species

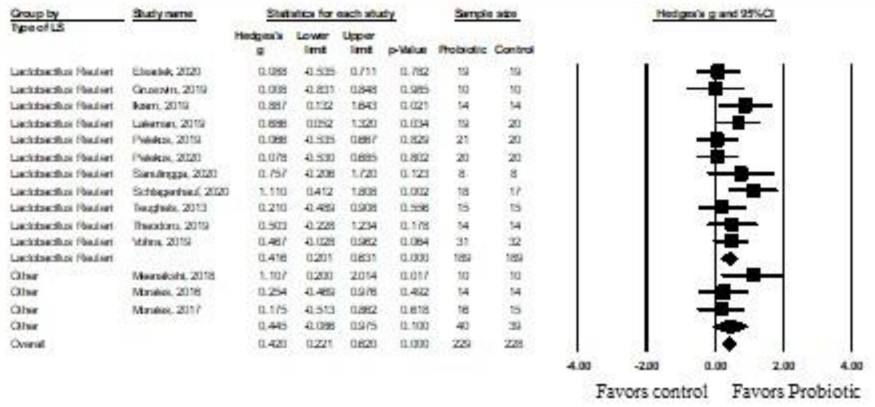

5.3. Treatment Duration

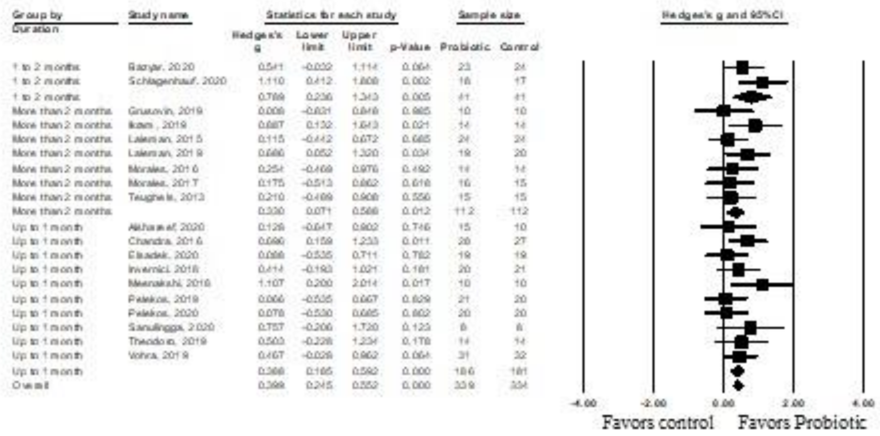

5.4. Mode of Probiotic Delivery

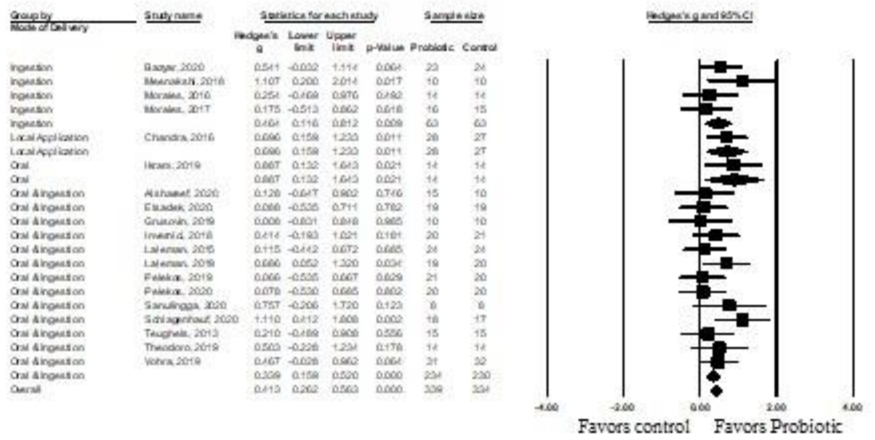

5.5. Oral Hygiene Instructions

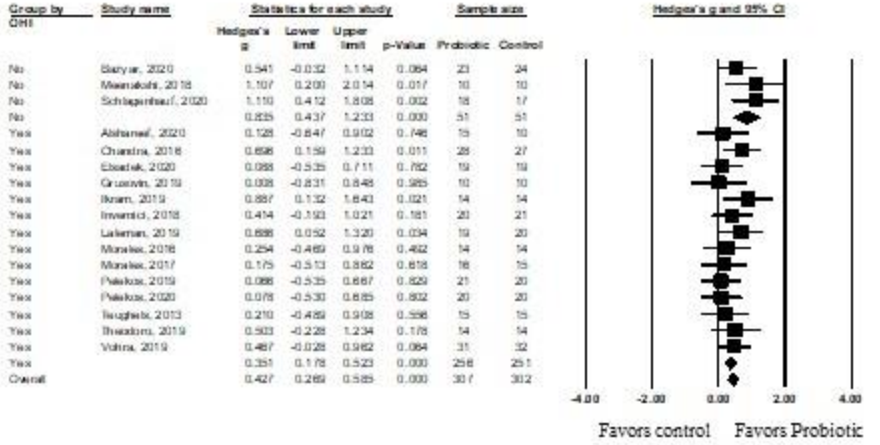

5.6. Disease Severity

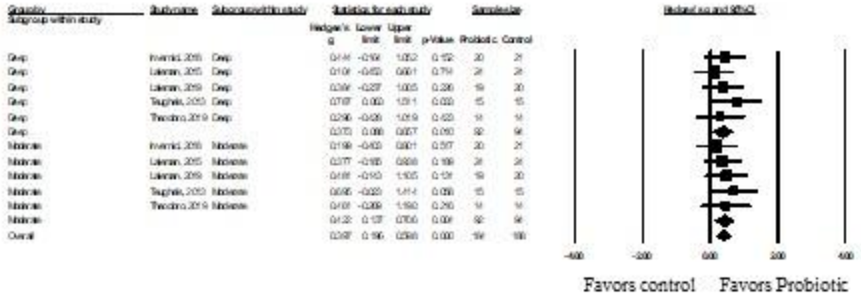

5.7. Comparison between Probiotics to Antibiotics

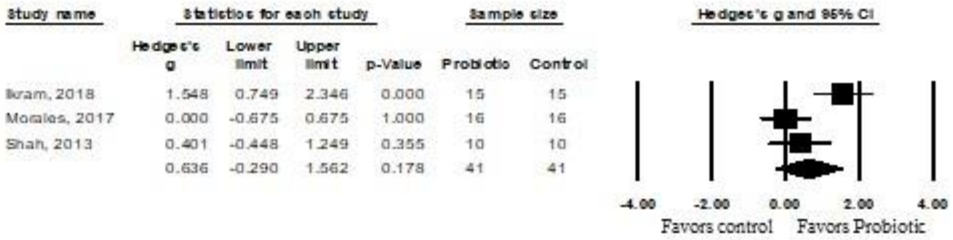

6. Bleeding on Probing (BOP)

6.1. Type of Periodontal Disease

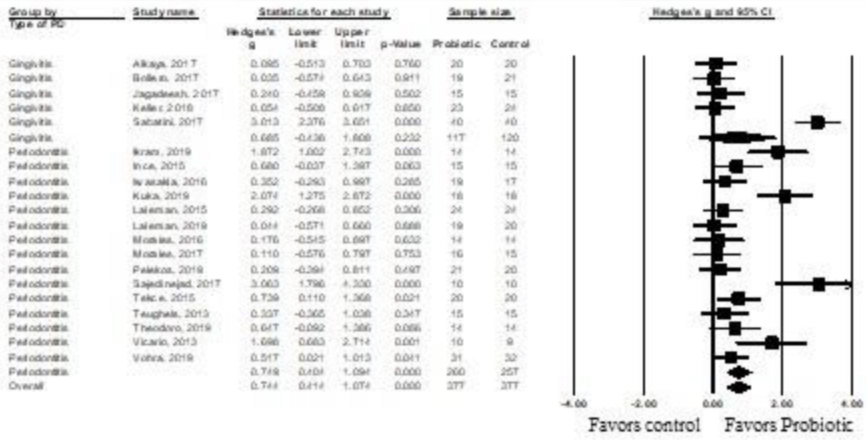

6.2. Type of Probiotic Strain

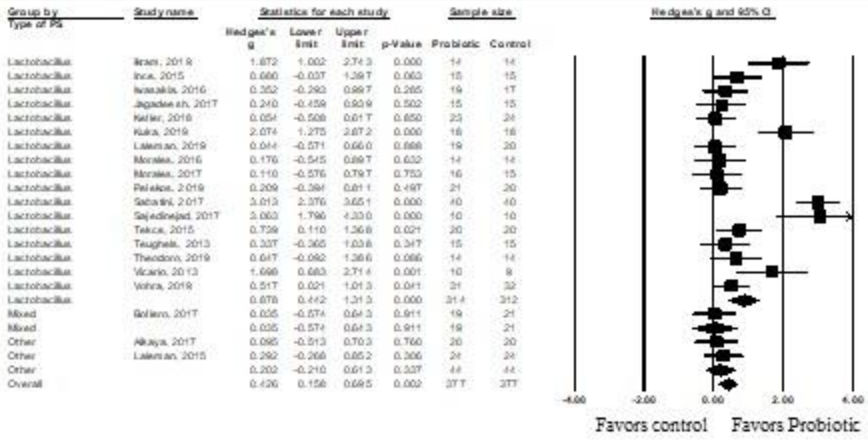

6.3. Type of *Lactobacillus* Species

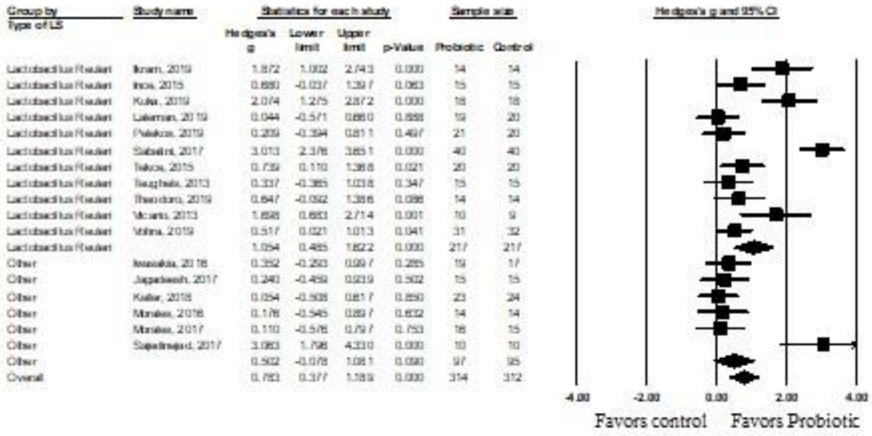

6.4. Treatment Duration

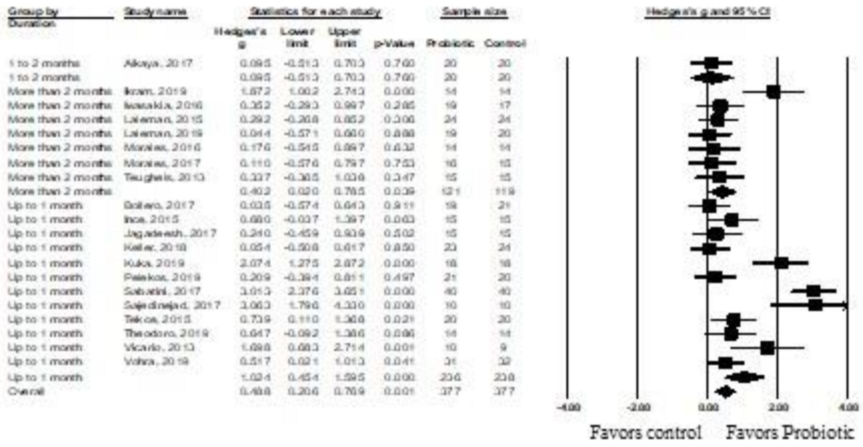

6.5. Mode of Probiotic Delivery

Group by Mode of Delivery	Study name	Hedges's g	Lower limit	Upper limit	p-Value	Probiotic	Control
Ingestion	Iwasakia, 2016	0.352	-0.293	0.997	0.285	19	17
Ingestion	Keller, 2018	0.054	-0.508	0.617	0.850	23	24
Ingestion	Morales, 2016	0.176	-0.545	0.897	0.632	14	14
Ingestion	Morales, 2017	0.110	-0.576	0.797	0.753	16	15
Ingestion	Sabatini, 2017	3.013	2.376	3.651	0.000	40	40
Ingestion		0.742	-0.391	1.876	0.199	112	110
Oral	Alkaya, 2017	0.095	-0.513	0.703	0.760	20	20
Oral	Bollero, 2017	0.035	-0.574	0.643	0.911	19	21
Oral	Ikram, 2019	1.872	1.002	2.743	0.000	14	14
Oral	Sajedinejad, 2017	3.063	1.796	4.330	0.000	10	10
Oral		1.166	-0.037	2.370	0.057	63	65
Oral & Ingestion	Ince, 2015	0.680	-0.037	1.397	0.063	15	15
Oral & Ingestion	Jagadeesh, 2017	0.240	-0.459	0.939	0.502	15	15
Oral & Ingestion	Kuka, 2019	2.074	1.275	2.872	0.000	18	18
Oral & Ingestion	Laleman, 2015	0.292	-0.268	0.852	0.306	24	24
Oral & Ingestion	Laleman, 2019	0.044	-0.571	0.660	0.888	19	20
Oral & Ingestion	Pelekos, 2019	0.209	-0.394	0.811	0.497	21	20
Oral & Ingestion	Tekce, 2015	0.739	0.110	1.368	0.021	20	20
Oral & Ingestion	Teughels, 2013	0.337	-0.365	1.038	0.347	15	15
Oral & Ingestion	Theodoro, 2019	0.647	-0.092	1.386	0.088	14	14
Oral & Ingestion	Vicario, 2013	1.698	0.683	2.714	0.001	10	9
Oral & Ingestion	Vohra, 2019	0.517	0.021	1.013	0.041	31	32
Oral & Ingestion		0.616	0.296	0.936	0.000	202	202
Overall		0.659	0.360	0.957	0.000	377	377

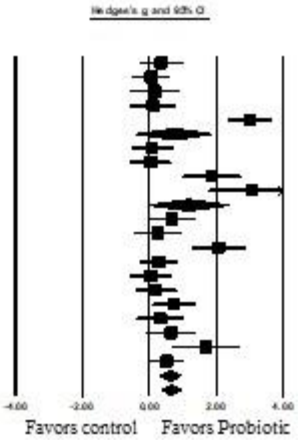

6.6. Oral Hygiene Instructions

Group by OHI	Study name	Hedges's g	Lower limit	Upper limit	p-Value	Probiotic	Control
No	Keller, 2018	0.054	-0.508	0.817	0.850	23	24
No		0.054	-0.508	0.817	0.850	23	24
Yes	Alkaya, 2017	0.095	-0.513	0.703	0.760	20	20
Yes	Ikram, 2019	1.872	1.002	2.743	0.000	14	14
Yes	Ince, 2015	0.680	-0.037	1.397	0.063	15	15
Yes	Kuka, 2019	2.074	1.275	2.872	0.000	18	18
Yes	Laleman, 2019	0.044	-0.571	0.660	0.888	19	20
Yes	Morales, 2016	0.176	-0.545	0.897	0.632	14	14
Yes	Morales, 2017	0.110	-0.576	0.797	0.753	16	15
Yes	Pelekos, 2019	0.209	-0.394	0.811	0.497	21	20
Yes	Sabatini, 2017	3.013	2.376	3.651	0.000	40	40
Yes	Sajedinejad, 2017	3.063	1.796	4.330	0.000	10	10
Yes	Tekce, 2015	0.739	0.110	1.368	0.021	20	20
Yes	Teughels, 2013	0.337	-0.365	1.038	0.347	15	15
Yes	Theodoro, 2019	0.647	-0.092	1.386	0.088	14	14
Yes	Vicario, 2013	1.698	0.683	2.714	0.001	10	9
Yes	Vohra, 2019	0.517	0.021	1.013	0.041	31	32
Yes		0.966	0.478	1.454	0.000	277	276
Overall		0.574	0.206	0.943	0.002	300	300

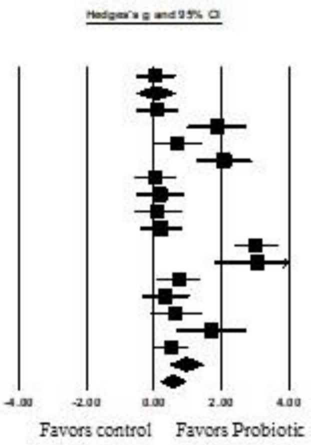

7. Gingival Crevicular Fluid (GCF)

7.1. Type of Periodontal Disease

Group by Type of PD	Study name	Hedges's g	Lower limit	Upper limit	p-Value	Probiotic	Control
Gingivitis	Keller, 2018	0.781	0.197	1.365	0.009	23	24
Gingivitis	Twetman, 2009	0.360	-0.405	1.126	0.356	13	12
Gingivitis		0.626	0.162	1.091	0.008	36	36
Periodontitis	Ince, 2015	0.329	-0.372	1.030	0.358	15	15
Periodontitis	Kuka, 2019	0.663	0.006	1.319	0.048	18	18
Periodontitis		0.507	0.027	0.986	0.038	33	33
Overall		0.568	0.235	0.902	0.001	69	69

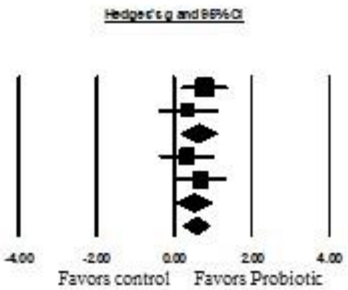

Supplemental Figure S1. Subgroup Analysis – Forest plot of the Hedge's g SMD comparing the effects of probiotic supplementation to control groups on clinical parameters using a random effects model: 1. Plaque Index (PlI); 2. Mean Plaque Percentage (MPP); 3. Gingival Index (GI); 4. Probing Pocket Depth (PPD); 5. Clinical Attachment Level (CAL); 6. Bleeding on Probing (BOP); 7. Gingival Crevicular Fluid (GCF).
